# Calcium Signaling and Meiotic Exit at Fertilization in *Xenopus* Egg

**DOI:** 10.3390/ijms151018659

**Published:** 2014-10-15

**Authors:** Alexander A. Tokmakov, Vasily E. Stefanov, Tetsushi Iwasaki, Ken-Ichi Sato, Yasuo Fukami

**Affiliations:** 1 Research Center for Environmental Genomics, Kobe University, Kobe 657-8501, Japan; E-Mails: tiwasaki@kobe-u.ac.jp (T.I.); yfukami@kobe-u.ac.jp (Y.F.); 2 Department of Biochemistry, St. Petersburg University, St. Petersburg 199034, Russia; E-Mail: stefanov@bio.pu.ru; 3 Department of Molecular Biosciences, Kyoto Sangyo University, Kyoto 603-8555, Japan; E-Mail: kksato@cc.kyoto-su.ac.jp

**Keywords:** eggs, fertilization, activation, calcium, *Xenopus laevis*

## Abstract

Calcium is a universal messenger that mediates egg activation at fertilization in all sexually reproducing species studied. However, signaling pathways leading to calcium generation and the mechanisms of calcium-induced exit from meiotic arrest vary substantially among species. Here, we review the pathways of calcium signaling and the mechanisms of meiotic exit at fertilization in the eggs of the established developmental model, African clawed frog, *Xenopus laevis*. We also discuss calcium involvement in the early fertilization-induced events in *Xenopus* egg, such as membrane depolarization, the increase in intracellular pH, cortical granule exocytosis, cortical contraction, contraction wave, cortical rotation, reformation of the nuclear envelope, sperm chromatin decondensation and sister chromatid segregation.

## 1. Introduction

Molecular mechanisms of sperm-egg interaction differ greatly among sexually reproducing biological species. However, some events of fertilization-induced egg activation are highly conserved. One of them, the generation of the calcium wave or calcium oscillations in the egg cytoplasm, was found to be universal. The calcium signal represents a main early event of fertilization-induced egg activation observed in all species studied. It is a prerequisite for subsequent fertilization events, such as the block to polyspermy, exit from meiotic arrest, nuclear reformation,* etc.* Notably, significant differences exist between the generation pathways, spatiotemporal patterns and downstream effectors of the fertilization calcium signal in different species. This review summarizes in a concise form our current knowledge about different aspects of calcium signaling at fertilization in the eggs of African clawed frog *Xenopus laevis*.

Frog oocytes and eggs have been widely used in studies of meiotic progression and fertilization. Many of the control mechanisms that operate in maturing oocytes, fertilized eggs and early embryos have been first established in the frog model*.* A number of pioneering studies, concerning involvement of calcium in egg fertilization, activation and meiotic exit, have been carried out using *Xenopus* eggs. Here, we also refer occasionally to other biological species; however, a comprehensive discussion of calcium signaling at fertilization in different species is outside the scope of this paper.

## 2. *Xenopus* Eggs before Fertilization

**Figure 1:**
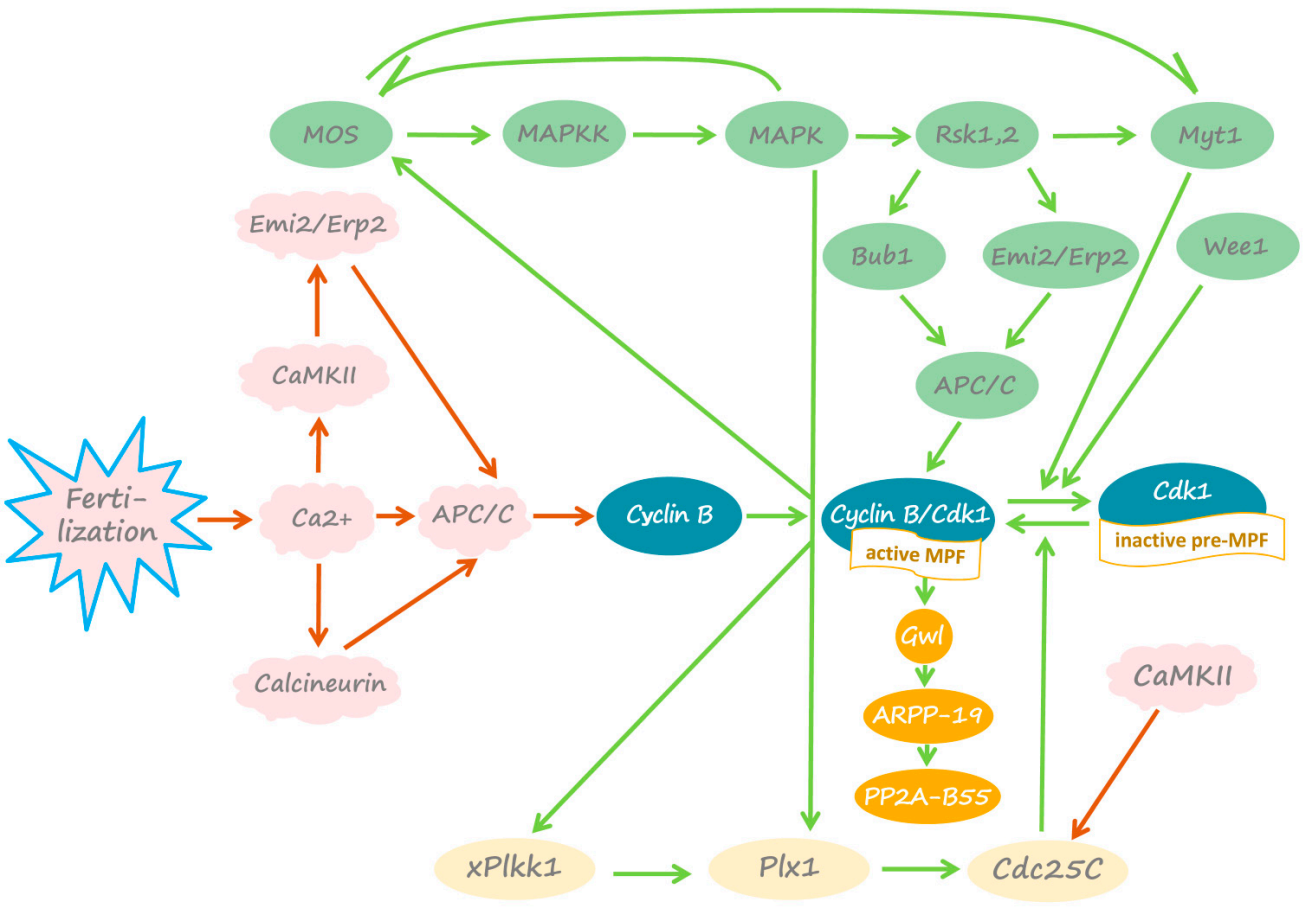
Signaling pathways in metaphase-arrested and fertilized *Xenopus* eggs. Molecular components of the signaling pathways operating in mature metaphase-arrested eggs and the factors involved in fertilization-induced exit from meiotic metaphase arrest are shown connected by green and red arrows, respectively. Detailed explanations are provided in the text (see Section 2 and Section 4).

To better understand the physiological significance of the fertilization-induced calcium signal, it is important to characterize the state of eggs prior to fertilization. In all sexually reproducing organisms, including *Xenopus*, oocytes undergo meiotic reduction divisions to yield a haploid content of chromosomes. Before that, immature fully-grown fertilization-incompetent *Xenopus* oocytes reside in the frog ovaries arrested in the prophase of the first meiotic division. The steroid hormone, progesterone, released from surrounding follicle cells, induces oocyte transition from prophase I to metaphase II in the process of meiotic maturation. In frogs, the term “egg” is conventionally used for the ovulated mature oocytes arrested in metaphase II. The cytoplasmic activity from eggs that causes complete maturation upon injection into immature oocytes was originally defined by Masui and Markert as a maturation promoting factor (MPF) [1]. It consists of cyclin B and cyclin-dependent protein kinase Cdk1 (Figure 1). The major bulk of Cdk1 in immature oocytes is present in a free inactive monomeric form, and some part of Cdk1 is stored as an inactive complex, called pre-MPF. Catalytic activity of Cdk1 in the oocytes is inhibited by phosphorylation on Thr 14 and Tyr 15 by the inhibitory kinase, Myt1 [2]. Another Cdk1-inhibitory kinase, Wee1, is not expressed in immature *Xenopus* oocytes and starts to accumulate at meiosis I exit [3,4]. The MPF-activating phosphatase Cdc25C is also inhibited in the immature oocytes by direct phosphorylation on Ser 287 [5]. In addition, the absence of an active cytostatic factor (CSF), defined as an activity that causes metaphase arrest in frog eggs [1], is also crucial for maintaining prophase I arrest. Although the exact molecular composition of CSF is not established, it was found that the proto-oncogenic protein kinase, Mos, which is present only during meiosis and disappears after fertilization [6], and the active MAPK pathway represent its major components [7,8,9].

After the completion of maturation, ovulated fertilization-competent *Xenopus* eggs are arrested in the metaphase of the second meiotic division due to the high activity of MPF and CSF. Cyclin B, synthesized during maturation, directly binds and activates Cdk1 kinase, whereas newly synthesized Mos protein supports the high activity of the MAPK cascade. The meiotic arrest allows eggs to await fertilization, preventing parthenogenetic continuation of cell cycles after meiosis. A number of interlocking feedback loops contribute to the stability of metaphase II arrest in mature eggs. Most notably, the MAPK cascades of CSF and MPF are embedded in a loop of positive feedback (Figure 1). Active MPF increases Mos stability by direct phosphorylation on Ser 3 [10]. In addition, MPF also upregulates Mos synthesis by enhancing polyadenylation of maternal *mos* mRNA [11]. On the other hand, MAPK, activated in the presence of Mos, phosphorylates and activates the downstream target protein kinase, Rsk, which phosphorylates and downregulates the Cdk1-inhibitory kinase, Myt1 (Figure 1) [2,12]. Another Cdk1-inactivating kinase, Wee1, is also inhibited in metaphase-arrested eggs via a phosphorylation-dependent mechanism [13]. Furthermore, Rsk directly phosphorylates and activates Emi2/Erp1 and Bub1 proteins, the inhibitors of the APC/C ubiquitin ligase, controlling cyclin B degradation [14,15,16]. Emi2/Erp1 protein is not expressed in prophase oocytes, but it accumulates in mature metaphase-arrested eggs, due to cytoplasmic polyadenylation and translational unmasking of its mRNA [17]. This protein was identified as a pivotal component of CSF required to maintain meiotic metaphase arrest [18,19]. The phosphorylated inhibitor proteins downregulate the APC/C by sequestering the Cdc20 activator subunit of the ligase [20]. In addition, both Cdk1 and MAPK activate the polo-like protein kinase pathway, including *Xenopus* polo-like kinase kinase xPlkk1 and polo-like kinase Plx1 (Figure 1) [21,22]. This pathway is linked to upregulation of the MPF-activating phosphatase Cdc25C (Figure 1) [23,24]. Some other mechanisms, such as Mos-stimulated polyadenylation of cyclin mRNA, also contribute to maintaining high MPF activity [25]. Furthermore, a protein synthesis-dependent loop of positive feedback in the MAPK cascade enhances Mos accumulation by promoting cytoplasmic polyadenylation of *mos* mRNA [11] and increasing the stability of Mos protein through its direct phosphorylation on Ser 3 [26].

Recently, it was found that high MPF activity in eukaryotic meiotic eggs and mitotic somatic cells involves the suppression of the major anti-Cdk1 phosphatase PP2A-B55, which dephosphorylates Cdk1-phosphorylated substrates [27]. This event is mediated by the Greatwall kinase (Gwl) activated downstream of Cdk1/cyclin B [28,29,30]. Activated Gwl directly phosphorylates two small related proteins of around 20 kDa, ARPP-19 and/or a-endosulfine, which then interact with and inhibit PP2A-B55 phosphatase (Figure 1) [31,32]. It was further demonstrated that the Gwl/ARPP19/PP2A-B55 module behaves as a major component of the MPF auto-amplification loop contributing to MPF activation in maturing *Xenopus* oocytes [33,34].

Thus, multiple interlocking loops of positive feedback promote and stabilize meiotic metaphase arrest in *Xenopus* eggs before fertilization (Figure 1). Fertilization triggers the disruption of the positive feedback between the two major determinants of the meiotic arrest, CSF and MPF, via calcium-dependent mechanisms, as explained further. Notably, eggs from different species are arrested at different stages of the meiotic cell cycle before fertilization (reviewed in [35,36]). However, the sperm-triggered calcium transient universally activates eggs and releases them from the meiotic arrest. The elevation of intracellular calcium is necessary and sufficient for egg activation.

## 3. Generation of Calcium Transient in *Xenopus* Egg at Fertilization

Although calcium universally mediates egg activation in different species, the upstream pathways by which sperm triggers the calcium signal within egg vary substantially among species. Currently, two opposing mechanisms of fertilization-induced elevation of intracellular calcium concentration have been elucidated. The sperm factor-mediated mechanism was found to trigger the calcium signal via a soluble factor released after gamete fusion from sperm into egg in mammals. For instance, a series of calcium oscillations is initiated by the sperm-specific phospholipase C, PLCζ, which is introduced by sperm into mouse eggs [37,38,39]. In some other species, the membrane receptor-mediated mechanism was proposed to account for the initiation of intracellular signaling after interaction between sperm ligand(s) and egg receptor(s). Fertilization in *Xenopus laevis*, as well as in sea urchin, ascidians, starfish, zebrafish,* etc.*, was found to involve membrane-originated tyrosine kinase-mediated generation of the calcium signal in eggs (reviewed in [40,41]). Notably, the two opposing mechanisms may not be necessarily mutually exclusive, and it has been suggested that *Xenopus* sperm may contain some biologically active compounds that contribute to egg activation. As a result of intracellular calcium release, its concentration in fertilized *Xenopus* eggs increases several fold from ~200 nM to above 1 µM within 5 min and returns to the pre-activation level within ~20 min after fertilization [42,43]. The calcium transient in fertilized *Xenopus* eggs, as well as that in the eggs of fish, sea urchin, jellyfish,* etc.*, represents a single calcium wave, propagating from the sperm entry point, within several minutes after fertilization. However, multiple calcium oscillations sustained for a much longer time were observed in the fertilized eggs of some other species, including mammals, ascidians, mollusks,* etc.* (reviewed in [36]). The calcium wave in fertilized *Xenopus* eggs travels from the animal to vegetal hemisphere of the egg, propagating at a rate of approximately 10 microns/s [42]. The apparent delay between the initial sperm-induced membrane depolarization, which can be considered as the start of fertilization, and initiation of the calcium wave at the sperm entry site was estimated to be approximately 1 min [42]. A delay of this magnitude strongly suggests that the fertilization-induced calcium signal is originated in the egg after a series of time-consuming intracellular events. These events were successfully delineated in recent studies.

The initial steps of sperm-egg interaction still remain obscure, and putative sperm ligand(s) and egg receptor(s) in the plasma membrane are yet to be identified. A plausible candidate for the egg receptor at fertilization in *Xenopus* can be the uroplakin III/uroplakin 1b complex [44,45]; however, surface-mediated activation of multiple receptor proteins in the egg membrane also cannot be ruled out. Involvement of the GTP-binding proteins, such as heterotrimeric G-proteins, was implicated by the fact that egg activation can be induced by the injection of the non-hydrolyzable GTP analog, GTPγS [46,47]. However, this was challenged later by the finding that an antibody against G_αq_ family G-proteins did not inhibit a calcium rise at fertilization, suggesting nonspecific effects of GTPγS [48]. Considering that no other type of G-protein α subunit can activate G-protein-sensitive PLCβ [49,50,51], this finding argues against a role for G-protein α subunits in the calcium rise at fertilization. In addition, it was shown that calcium release at fertilization of frog eggs is not inhibited by pertussis toxin, suggesting that βγ subunits from G_i_ or G_o_ are not required for this process [46]. Still, involvement of the pertussis toxin-insensitive βγ subunits was not investigated in detail, and it cannot be ruled out completely.

**Figure 2:**
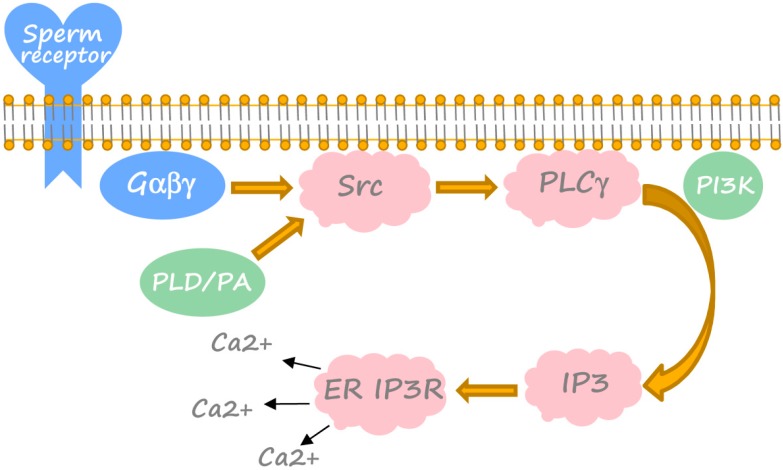
Fertilization-induced cascade of intracellular calcium release in *Xenopus* eggs. Major established constituents, crucial accessory factors and unknown putative components of the cascade are shown in pink, green and blue, correspondingly. Detailed explanations are provided in the text (see Section 3).

A number of studies established the sequential activation of Src family kinases, PLCγ and IP3 receptor of the endoplasmic reticulum as the early universal events of fertilization-induced egg activation (Figure 2) (reviewed in [52,53,54,55]). These events precede the calcium transient. The signaling pathway leading to the calcium rise was delineated in *Xenopus* eggs and egg extracts using mainly an inhibitor-based approach [48,56,57,58,59]. It was shown that, although Src kinase does not play a significant role in *Xenopus* oocyte maturation and meiotic arrest [60], it is indispensable for fertilization-induced egg activation, as demonstrated with the use of pharmacological inhibitors of Src-family kinases [56,57]. Activation of Src kinase can be detected in *Xenopus* eggs within 1 min after fertilization [61]. In parallel, PLCγ becomes tyrosine-phosphorylated, translocated from the cytoplasm to the membrane, associated with Src and several-fold activated [57,58,62]. Then, activated PLCγ produces IP3 and DAG by hydrolysis of PIP2. A role for SH2-domain mediated activation of PLCγ in *Xenopus* egg activation is unlikely, since injecting PLCγ SH2 domains does not inhibit the calcium rise at fertilization [63]. Instead, the PH domain binding to the membrane PIP2/PIP3 may be involved in the process [54]. This suggests the engagement of the PIP3-generating enzyme, PI3 kinase (Figure 2). Its pharmacological inhibition was found to suppress fertilization-induced PLCγ activation and calcium release [59].

The IP3 mass increase in *Xenopus* eggs starts 1 min after fertilization and reaches more than four-fold excess over the basal state in about 5 min [57,64]. IP3 triggers initial calcium release from the IP3-receptor-operated calcium ER stores at the sperm binding site: an antibody against the type I IP3 receptor blocks calcium release in fertilized *Xenopus* eggs [48]. However, following self-propagation of the calcium wave in the egg involves, most probably, two processes: IP3-induced calcium release (IICR) and calcium-induced calcium release (CICR) [65,66,67]. Notably,* Xenopus* eggs acquire the capacity to generate the fertilization-specific calcium transient during oocyte maturation; injection of IP3 in eggs results in a slower and more prolonged calcium wave than that in oocytes [68]. The major events of calcium signaling differentiation during maturation include internalization of the plasma membrane calcium ATPase, loss of store-operated calcium entry and spatial reorganization of IP3 receptors in the ER membrane [68,69,70,71]. Recently, computational modeling demonstrated that jointly, these factors can account for the observed changes in calcium signaling wave propagation [72].

Notably, evidence has been provided that phospholipase D (PLD) and phosphatidic acid (PA) may act as upstream regulators of Src and PLCγ in *Xenopus* eggs fertilization (Figure 2) [73]. Indeed, fertilization elevates PA levels within 1 min of fertilization, and PA addition to *Xenopus* eggs stimulates Src and tyrosine phosphorylation of PLCγ, increases the intracellular mass of IP3 and induces fertilization events that can be blocked by an IP3 receptor inhibitor or the calcium chelator, BAPTA [74]. PLD hydrolizes phosphatidylcholine to produce PA and choline, whose level also increases at fertilization [73]. Pharmacological inhibition of PLD was found to suppress Src and PLCγ activation, calcium release and other fertilization-induced events [74]. Molecular species analysis and mass measurements suggest that sperm activates the PLD1b isoform to elevate PA [74].

The calcium transient initiates a plethora of biochemical and cellular events in fertilized *Xenopus* eggs, such as exit from meiotic arrest, membrane depolarization, exocytosis of cortical granules, elevation of fertilization envelope, cortical contraction, cortical rotation, sperm decondensation, pronuclear formation,* etc.* The following sections of the manuscript reveal these events in more detail.

## 4. Fertilization-Induced Meiotic Exit

The major event of *Xenopus* egg activation following fertilization is calcium-induced exit from meiotic metaphase II arrest and cell cycle resumption. Fertilization triggers MPF inactivation before Mos degradation and MAPK pathway inactivation, pinpointing MPF as an early target. Calcium-dependent degradation of cyclin B occurs within 15 min of fertilization. It causes the disruption of positive feedback between MPF and CSF and Mos protein degradation within 30–40 min after fertilization [75]. The calcium/calmodulin-dependent protein kinase, CaMKII, was found to mediate the effect of calcium on cyclin degradation. Inhibition of CaMKII prevents cyclin degradation and exit from metaphase arrest after calcium addition, whereas microinjection of constitutively active CaMKII into unfertilized *Xenopus* eggs inactivates Cdk1 and releases the eggs from the CSF arrest in the absence of the calcium transient [76]. It was found that CaMKII conveys fertilization-induced cyclin degradation and meiotic exit in *Xenopus* eggs via the APC/C inhibitor, Emi2/Erp1 (Figure 1). CaMKII directly phosphorylates Emi2/Erp1 on Thr 195 to create a polo-box binding site for the polo-like kinase Plx1. Binding of Plx1 promotes the secondary phosphorylation of Emi2/Erp1 by Plx1 on Ser 33 and Ser 38 at the site of the phosphorylation-dependent degradation signal [77,78]. The signal is then recognized by the SCF1 (SKP2-cullin1-F-box protein)-E3 ubiquitin ligase complex targeting Emi2/Erp1 for 26S proteasome-mediated degradation [18,78,79,80,81]. Degradation of Emi2/Erp1 leads to APC/C activation and cyclin B ubiquitination, directing Cdk1/cyclin B complex to the 26S proteasome. Finally, proteasome-dependent dissociation of the complex, followed by cyclin B degradation, inactivates MPF [82].

In addition to targeting the protein degradation machinery, activated CaMKII inhibits Cdc25C phosphatase by direct phosphorylation on Ser 287 (Figure 1) [83]. This phosphorylation is suppressed in unfertilized M phase-arrested *Xenopus* eggs. Calcium-induced inhibition of Cdc25C activity in the fertilized eggs increases phosphorylation levels of Thr 14 and Tyr 15 in Cdk1, thereby inhibiting its kinase activity.

Independently of CaMKII, the fertilization-induced calcium transient activates the calcium/calmodulin-dependent serine/threonine protein phosphatase calcineurin (PP2B) (Figure 1). Calcineurin activity is essential for overcoming meiotic arrest. Inhibition or depletion of calcineurin in *Xenopus* egg extracts delays cyclin B destruction, Cdk1 inactivation and the global dephosphorylation of M-phase-specific phosphoproteins in response to calcium [84,85]. Furthermore, inhibition of calcineurin in unfertilized eggs prevents meiotic exit upon egg activation [85]. It was reported that calcineurin is rapidly and transiently activated immediately after calcium addition to CSF-arrested *Xenopus* egg extracts [85]. Then, calcineurin dephosphorylates the APC/C activator Cdc20 and the core APC/C component Apc3 (also known as Cdc27). These dephosphorylation events have been suggested to contribute to APC/C activation via a yet unknown mechanism. Previously, evidence was provided that phosphorylation of Cdc20 inhibits its ability to activate the APC/C [86]. It remains to be clarified whether APC/C activation depends on dephosphorylation of its subunits by calcineurin. Furthermore, the role of calcineurin in the meiotic activation of PP2A-B55 phosphatase requires investigation. The findings obtained using *Xenopus* egg extracts demonstrate that PP2A-B55 phosphatase, suppressed in metaphase II by the high activity of Gwl kinase (see Section 2), becomes activated when Cdk1 is inactivated and the CSF-arrested extracts are released into interphase [27,87].

Thus, the fertilization-induced calcium transient independently activates CaMKII and calcineurin. They both contribute to inhibition of Cdk1 activity in fertilized *Xenopus* eggs. Cdk1 inactivation triggers the disruption of positive feedback between MPF and CSF. Mos protein is dephosphorylated at Ser 3, a site of direct phosphorylation by Cdk1, and degraded by the *N*-terminal Pro2-dependent ubiquitin pathway [88]. In addition, EDEN-directed deadenylation of *mos* mRNA effectively suppresses Mos translation after fertilization [89,90]. Mos degradation leads to shutdown of the MAPK cascade and complete CSF inactivation. It was suggested that the calcium-dependent cysteine protease, calpain, may also play a role in fertilization-induced Mos degradation [91]. However, this idea was challenged by the finding that calpain is capable of degrading Mos* in vitro* only at supraphysiological concentrations [92].

Of note, the involvement of CaMKII and calcineurin in egg activation is not evolutionary conserved. Mature eggs of many invertebrate species arrest at metaphase I, and their genomes do not encode the CaMKII target protein, Erp1/Emi2. It was shown that the two phosphatases, calcineurin and PP2A, but not CaMKII, are required for normal egg activation during fertilization in ascidians [93]. On the other hand, cell cycle resumption in fertilized mammalian (mouse) eggs relies solely on CaMKII [20,94,95]. It was hypothesized that during animal evolution, the mechanism of egg activation evolved from the phosphatase-dependent process to the protein kinase-dependent one, with *Xenopus* being an intermediate species involving both of them [93]. Analysis of egg activation signaling pathways in various animal species is necessary to validate this hypothesis.

## 5. Other Effects of Calcium in Fertilized *Xenopus* Egg

The fertilization-induced calcium transient is responsible for several biochemical and cellular events of *Xenopus* egg activation. Membrane depolarization, exocytosis of cortical granules, elevation of fertilization envelope, sperm decondensation, activation-associated increase in intracellular pH and pronuclear formation were found to be inhibited or delayed in the activated eggs by prior microinjection of the calcium chelator, BAPTA [96,97]. Furthermore, BAPTA or heparin, an IP3 receptor antagonist, prevented the calcium-dependent fertilization events, such as gravitational rotation, contraction wave and cleavage furrow formation [98,99]. Further, it was shown that calcium triggers sister chromatid segregation in the extracts prepared from unfertilized *Xenopus* eggs,* i.e.*, CSF-arrested extracts [100,101]. At present, the pathways leading to these calcium-mediated events are poorly understood.

It is thought that activation of protein kinase C (PKC) due to elevation of DAG and intracellular calcium is responsible for cortical granule exocytosis, cortical contraction, reformation of the nuclear envelope and sperm chromatin decondensation. Indeed, a wave of PKC activation accompanies the calcium transient after fertilization of *Xenopus* egg [102]. Activators of PKC are able to trigger cortical granule exocytosis, cortical contraction and cleavage furrow formation in unfertilized *Xenopus* eggs [103]. Furthermore, nuclear lamina disassembly of permeabilized sperm nuclei in sea urchin egg extracts was found to be a result of lamin B phosphorylation, which is reversibly inhibited by PKC-specific inhibitors, but not by inhibitors of PKA, Cdk1 or CaMKII [104]. As lamin B phosphorylation and solubilization precede chromatin decondensation, PKC inhibition also prevents sperm decondensation.

Importantly, the DAG increase at fertilization does not originate from PIP2 hydrolysis by PLCγ. It was reported that the increase in DAG mass and translocation of PKCα and PKCβ to a membrane fraction occur in *Xenopus* egg in about 7 min after fertilization [73]. Thus, the DAG increase at fertilization occurs later than that of IP3, and besides, it is approximately 280-times greater. Combined with the facts that choline mass also increases at fertilization and that the total choline increase is comparable to that of DAG, these data suggest that most of DAG in fertilized *Xenopus* eggs is produced by activated PLD, which hydrolyzes phosphatidylcholine to PA and choline, and PA is further converted to DAG [54,74,105].

Although PKC is widely involved in calcium-mediated fertilization signaling, many events of egg activation do not require its participation. For instance, the depolarizing fertilization potential, which provides the electric block to polyspermy, is directly mediated by calcium-activated Cl^−^ channels. These channels become open due to an increase in intracellular calcium triggered by IP3-induced calcium release [43,106,107]. Furthermore, PKC activators, unlike calcium ionophores, cannot mimic fertilization-induced increase in intracellular pH, suggesting that this increase is not related to PKC-dependent phosphorylation of the Na^+^/H^+^ exchanger in the plasma membrane [108]. Presently, the mechanism of fertilization-induced calcium-mediated increase in intracellular pH remains obscure.

Further, the fertilization-induced surface contraction wave (SCW) of pigmentation change at the egg cortex, which originates at the animal pole and proceeds to the vegetal pole, does not depend on PKC and relies, most probably, on Cdk1 activity. It was found that the contraction phase requires inactivation of MPF and is blocked when MPF activity is maintained at elevated levels [109]. Thus, SCW represents a local surface response to a wave of cytoplasmic MPF inactivation. It was hypothesized that the change in pigmentation is controlled by the phosphorylation of microtubule-associated proteins by Cdk1 [110].

Similarly, Cdk1 activity also governs cortical rotation, a fertilization-induced microtubule-mediated process that translocates the egg cortex relative to the cytoplasm, specifying the orientation of the embryonic dorso-ventral axis. Cortical rotation stops at mitosis I entry, at the time of MPF activation, accompanied by a visible SCW [110,111]. Cortical rotation can be prolonged if MPF activation does not occur, while injections of MPF can provoke ectopic SCW waves that arrest cortical rotation [111]. It was further demonstrated that cortical rotation is arrested not by MPF-dependent inhibition of molecular motors, such as kinesin-related proteins and dynein, but as a result of MPF-induced microtubule depolymerization [112]. The involvement of microtubule-associated proteins, whose activity is modulated by MPF-dependent phosphorylation, was suggested [113,114,115]; however, the exact mechanism of cortical rotation and its regulation by MPF still remain to be understood.

Another example of the PKC-independent egg activation event concerns sister chromatid segregation at anaphase. This event is induced by CaMKII. A constitutively active CaMKII mutant was found to trigger anaphase in the absence of the calcium transient [101,116]. It was further shown in activated *Xenopus* eggs and calcium-treated CSF-arrested egg extracts that segregation of sister chromatids at the second meiotic anaphase is controlled by the APC/Cdc20-dependent pathway [117], which is suppressed in MII-arrested eggs and becomes activated in fertilized eggs (for details, see Section 2 and Section 4 of this review). CaMKII and APC/C co-localize in mitotic spindles and centrosomes of mammalian cells, suggesting their functional connections [118]. A direct target of the activated APC/C in centromeres is securin, which is degraded in late metaphase via the ubiquitin-proteasome pathway. Non-degradable *Xenopus* securin prevents* in vitro* assembled spindles from separating sister chromatids at exit from mitosis in *Xenopus* egg extracts [119]. Destruction of securin unleashes the large cysteine endopeptidase, separase, which is held inactive by association with securin. It was reported that removal of an inhibitory phosphate is also necessary for full separase activation [120]. Finally, separase cleaves the kleisin subunit SCC1 of the ring-shaped multi-protein complex cohesion, which is responsible for holding sister chromatids together [121].

## 6. Conclusions and Perspectives

The calcium signal at fertilization has been exceptionally conserved through the course of evolution. It represents an early indispensable event of fertilization-induced egg activation. So far, the research on the molecular mechanisms of fertilization has been focused on several biological species. Among them, *Xenopus* *laevis* has served as a very useful model organism for studying the fertilization-induced calcium transient. *Xenopus* eggs are arrested in meiotic metaphase II awaiting fertilization. The multiple interlocking loops of positive feedback, particularly that between MPF and CSF, stabilize the meiotic metaphase arrest. Fertilization-induced elevation of intracellular calcium disrupts the feedback, activates eggs and releases them from meiotic arrest. The importance of the Src-PLCγ-IP3 pathway in the generation of the calcium transient at fertilization has been established. However, the initial steps of sperm-egg interaction upstream of this pathway are obscure. Most notably, the identity of the egg membrane-associated receptor for sperm remains elusive. The main event of *Xenopus* egg activation at fertilization is calcium-induced exit from meiotic metaphase arrest. The calcium transient independently activates CaMKII and calcineurin in *Xenopus* eggs. They both contribute to Cdk1 inhibition by activating the APC/C ubiquitin ligase via different mechanisms involving Emi2/Erp1, Apc3 and Cdc20 regulators of APC/C. Cdk1 inactivation triggers disruption of the major loop of the positive feedback between MPF and CSF, resulting in meiotic exit. The emerging involvement of PP2A-B55 phosphatase in these processes requires further investigation.

In addition, calcium orchestrates a plethora of biochemical and cellular events of *Xenopus* egg activation, such as membrane depolarization, increase in intracellular pH, cortical granule exocytosis, cortical contraction, contraction wave, cortical rotation, reformation of the nuclear envelope, sperm chromatin decondensation, sister chromatid segregation and some others. Little is known about the calcium-dependent mechanisms underlying these steps of egg activation. Their future investigation may potentially reveal novel molecular targets of calcium in eukaryotic cells. Thus, *Xenopus* eggs will remain a major biological model for fertilization and basic physiological studies, due to their high biological relevance, convenience of manipulation and biochemical tractability.
